# Neoplastic leptomeningitis presenting in a melanoma patient treated with dabrafenib (a ^V600E^BRAF inhibitor): a case report

**DOI:** 10.1186/1752-1947-6-131

**Published:** 2012-05-17

**Authors:** Ester Simeone, Eleonora De Maio, Fabio Sandomenico, Franco Fulciniti, Secondo Lastoria, Pasquale Aprea, Stefania Staibano, Vincenzo Montesarchio, Giuseppe Palmieri, Nicola Mozzillo, Paolo A Ascierto

**Affiliations:** 1 Istituto Nazionale Tumori, Fondazione “G. Pascale”, Napoli, Italy; 2 Institute of Pathology, University of Napoli “Federico II”, Napoli, Italy; 3 Unit of Medical Oncology, Ospedale Cotugno A.O.R.N. dei Colli, Napoli, Italy; 4 Institute of Biomolecular Chemistry, National Research Council (CNR), Sassari, Italy; 5 Unit of Medical Oncology and Innovative Therapy, Istituto Nazionale per lo Studio e la Cura dei Tumori “Fondazione G. Pascale”, Via Mariano Semmola, 80131, Napoli, Italy

## Abstract

****Introduction**:**

Leptomeningeal metastases are occurring at higher frequency in cancer patients. The prognosis of leptomeningeal metastases is poor and standard treatment, which includes radiotherapy and chemotherapy, is mostly ineffective. Melanoma represents one of the tumors with the highest incidence of leptomeningeal metastases. For such a disease, the BRAF inhibitors have recently been demonstrated to be effective on melanoma brain metastases harboring the ^V600E^BRAF mutation.

****Case presentation**:**

We report a case of a 39-year-old Italian woman with advanced melanoma with brain, lung and peritoneum metastases harboring the ^V600E^BRAF mutation. In August 2010 she was enrolled into the BRIM3 trial and after the randomization process she received dacarbazine. After two cycles, there was evidence of disease progression in her peritoneum and lung. For this reason, she was enrolled into another clinical trial with the GSK2118436 BRAF inhibitor, dabrafenib, as a second line of therapy. She had a partial response that was maintained until 13 weeks of treatment. In January 2011 she developed symptoms typical for brain metastases and received a diagnosis of leptomeningeal involvement of melanoma cells after an examination of her cerebral spinal fluid; magnetic resonance imaging was negative for meningitis or brain metastases. Analysis of her cerebral spinal fluid sample confirmed that the melanoma cells still carried the ^V600E^BRAF mutation. After a few days, our patient went into a coma and died.

****Conclusion**:**

Starting with a clinical case, we discuss the pathogenesis of leptomeningeal metastases and whether the leptomeninges may represent a sanctuary where melanoma cells may generate resistance and/or BRAF inhibitors cannot reach an adequate concentration for significant activity. We assess whether treatment with BRAF inhibitors in melanoma patients should be interrupted as soon as disease progression appears or continued beyond progression, through the administration of additional compounds.

## **Introduction**

The incidence of leptomeningeal metastases (LM) in cancer patients has increased, probably due to the achievement of prolonged survival. Both solid tumors (including breast, lung and gastrointestinal carcinomas as well as melanoma) and hematopoietic tumors (including lymphoma and leukemia) may induce LM formation [1]. The prognosis is poor and less than 10% of patients survive to 12 months [1,2]. The base of the brain and the cauda equina are the most prevalent sites of metastasis. Standard treatment, which includes radiotherapy to symptomatic sites and intrathecal chemotherapy, is mostly ineffective [3].

Recently, two important compounds changed the history of treatment for advanced melanoma: the anti- cytotoxic T-lymphocyte antigen 4 (CTLA4) monoclonal antibody [4,5] among unselected patients and the BRAF inhibitors (BRAFi) [6] among patients carrying a mutation at the valine 600 codon in the *BRAF* gene (^V600E^BRAF mutation). Although both seem to act on melanoma brain metastases [4,7], the BRAFi (vemurafenib, GSK2118436, dabrafenib) seem to be particularly effective on melanoma brain metastases harboring the ^V600E^BRAF mutation - which represents the most prevalent oncogenic variant in such a gene [7-9]. Moreover, a high concordance for ^V600E^BRAF mutation frequency between primary melanomas and correspondent brain metastases from the same patients has been recently reported by our group [10]. To date, two important studies are focusing on the treatment of melanoma brain metastases with BRAFi [11,12].

Here, we report the clinical case of a woman who developed LM disease during BRAFi treatment and discuss more general considerations about melanoma brain involvement.

## **Case presentation**

**Figure 1:**
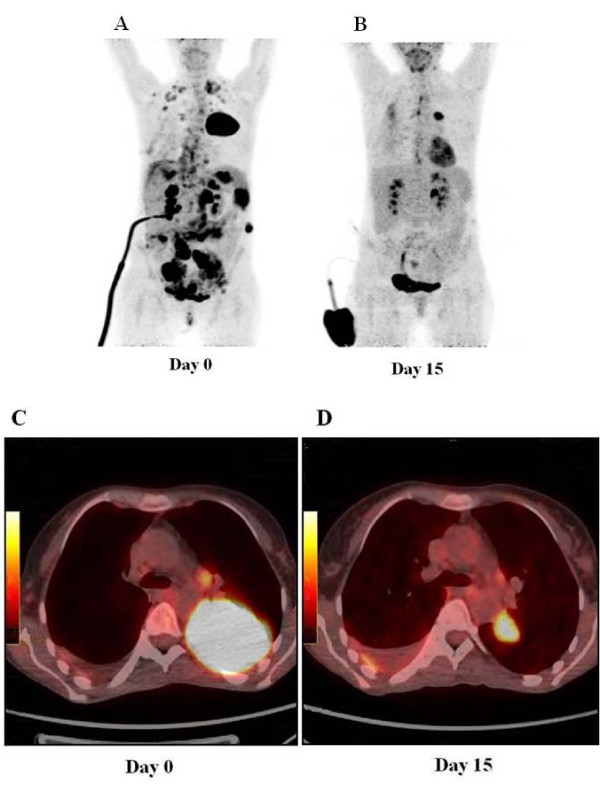
**Positron emission tomography scan evaluation. (A and C**) At baseline; **(B and D**) after 15 days.

**Figure 2:**
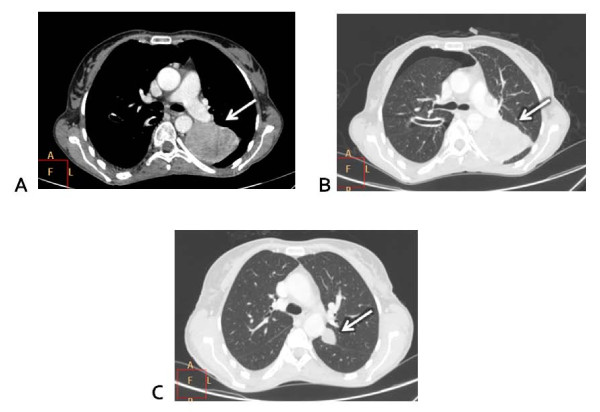
**Computed tomography scan evaluation. (A)** Baseline: great metastatic nodular lesion on the left lung (white arrow). **(B)** Baseline: lung parenchymal windows showing the great lesion on the left and a drainage tube for thoracentesis with residual pneumothorax (white arrow). **(C)** Week 12 under treatment: reduction of the great lung lesion with small residual nodule (white arrow).

**Figure 3:**
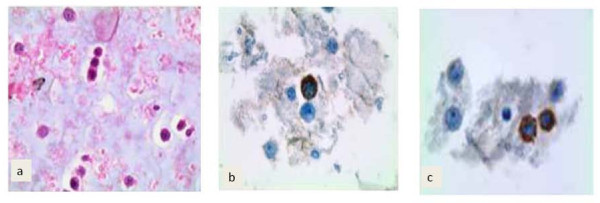
**Liquor sample evaluation. (A)** Hematoxylin and eosin staining; **(B)** HMB45 immunocytochemistry; **(C)** S100 immunocytochemistry.

A 39-year-old Italian woman, who received the diagnosis of cutaneous melanoma in 2005, was enrolled into the BRIM3 trial (vemurafenib versus dacarbazine [6]) in August 2010 after disease progression was ascertained with the detection of metastases in both her lung and peritoneum. Despite being positive for the ^V600E^BRAF mutation, she was randomized to receive dacarbazine. After two cycles, disease progression was registered, with the appearance of new peritoneal lesions associated with ascites and lung lesions associated with pleural effusion. Therefore, our patient was enrolled into another clinical trial with GSK2118436 BRAF inhibitor, dabrafenib, as a second line of therapy. After two weeks of treatment, the ascites and pleural effusion disappeared and her visceral lesions also reduced dramatically (Figure 1); this partial response was maintained over 13 weeks of treatment until the beginning of January 2011 (Figure 2), when a diagnosis of leptomeningeal involvement of the melanoma cells was inferred by a cerebral spinal fluid (CSF) examination - with magnetic resonance imaging negative for meningitis or brain metastases (Figure 3). Analysis of her CSF sample confirmed that the melanoma cells still carried the ^V600E^BRAF mutation (not shown). After a few days, our patient went into a coma and died.

## **Discussion**

To the best of our knowledge, there are no data available for the use of BRAFi in controlling LM from melanoma. The only clinical evidence provided for such types of drugs has been regarding their effectiveness on brain metastases [7-9]. Although unknown, the effect of BRAFi on LM may be due to different molecular mechanisms. Preclinical studies on such lesions showed the involvement of at least two important processes: angiogenesis and proliferation [13-18].

Reijneveld *et al*. demonstrated that neovascularization is important for the growth of LM in mice and in humans [13]. Systemic targeting of the vascular compartment may be a useful approach in novel therapeutic strategies for patients with LM. The identification of selective receptors on brain capillary endothelium and tumor cells, which facilitate tumor cell adhesion and metastasis formation at restricted sites, may represent an important therapeutic target. In particular, Brandsma *et al*. have indicated the potential importance of integrin expression by demonstrating that constitutive integrin activation on leukemic cells contributed to leptomeningeal leukemia [14]. In that case, authors attributed such findings to an increased integrin-mediated leukemic cell adhesion to the leptomeninges, which mostly involved β3 integrin as determined by *in vitro* assays on primary leptomeningeal cell layers [14]. The Ras-related guanosine triphosphatase protein, Rap-1, a protein that has been shown to be a key regulator of integrin activation in leukocytes, may be another interesting candidate [15-17]. This research could lead to the development of agents that efficiently block tumor cell adhesion in order to prevent LM progression. Küsters-Vandevelde *et al.* investigated the expression of activating mutations of the *GNAQ* gene in primary melanocytic tumors of the central nervous system (CNS) as well as the mutation status of *BRAFNRAS* and *HRAS* genes on LM [18]. The *GNAQ* mutations were found in uveal melanoma and primary melanocytic lesions of the CNS (mutations in codon 209 of *GNAQ* form an alternative route to mitogen-activated protein kinase (MAPK) activation), while mutations of *NRAS* and *BRAF* were detected in metastatic lesions of the CNS, including LM (no involvement of *HRAS* was observed) [18]. Our data indicated a frequency of 48% for cerebral ^V600^BRAF mutations, with a quite similar incidence rate of such BRAF variants among primary melanomas and corresponding brain metastases from the same patients [10]. This suggests that melanoma cells are unlikely to change their *BRAF* mutational status during the formation of brain metastasis. As a consequence, inhibitors of mutated *BRAF* are postulated to represent a therapeutic approach in LM from melanoma.

Despite indications that inhibitors of mutated *BRAF* may represent an effective therapeutic approach in melanoma brain metastases, our patient in this case showed responsiveness on visceral sites but dramatic disease progression to the leptomeninges during therapy with BRAFi.

From the biological point of view, this is consistent with the hypothesis that acquired resistance to BRAF inhibition may depend on the activation of alternative survival pathways, with no modification of the *BRAF* mutational status [19-24]. In particular, a large variety of induced alterations has been indicated to drive resistance to BRAFi. These include upregulation of the receptor tyrosine kinase effectors or activating alterations of *NRAS* or *PDGFRβ* genes [19]; switching among the three Raf isoforms with, especially, increased levels of the CRAF protein [20]; amplification of the *CCND1/Cyclin D1* gene or lack of phosphatase-and-tensin homologue function [21]; or mutations in the downstream gene for methyl ethyl ketone (MEK) [22]. Recently, two additional mechanisms of resistance to BRAFi have been described: activation of the *MAP3K8* gene, which encodes the COT MAPK pathway agonist (COT is able to activate downstream extracellular signal-regulated kinase protein through a MEK-dependent mechanism not requiring RAF-driven signals) [23], and induction of the alternative BRAF-independent insulin growth factor receptor 1- phosphatidylinositol-3-kinase (PI3K) signaling pathway with increased intracellular levels of the downstream Akt effector [24]. Nevertheless, although the leptomeninges may represent a special ‘sanctuary’ site for the development of resistance, a combination of response and resistance in specific sites as well as a sequential occurrence of an initial response and later tumor growth within metastases in the setting of BRAFi should be carefully taken into account.

From the clinical point of view, these findings open the discussion as to whether treatment with BRAFi should be interrupted as soon as progression appears or if a different treatment should be added to BRAFi-based therapy. Recently, a study indicated that the control of disease progression is not necessarily rapid under BRAFi treatment and, in a subset of patients with disease progression, continuation of treatment with BRAFi may be potentially beneficial [25]. These authors suggested the need for further study to evaluate the impact of post-progression treatment with the BRAFi [25]. In cases of a mixed response (complete or partial response in some lesions associated with progression in other sites), we propose that therapy with BRAFi should be continued and treatment with a new agent (for example, a MEK inhibitor, PI3K inhibitor or anti-CTLA4) should be added. This combination approach will surely represent the standard therapeutic strategy in the future [26]. Recently, a phase I study based on indications derived from preclinical models suggested that an upfront combination therapy, instead of a sequential administration of targeted compounds, may act as a very promising approach toward the reduction of the time to resistance [27].

In our case, we demonstrated that the ^V600E^BRAF mutation was still present in melanoma cells from our patient’s CSF. This could indicate that the disease progression to leptomeninges was due to the development of drug resistance in *BRAF* mutated cells (for example, through a switch to the CRAF signaling pathway) and not to the appearance of a different melanoma brain clone.

## **Conclusions**

The occurrence of LM has to be considered as a possible event during treatment with BRAFi in patients with melanoma. Although further studies based on more appropriate samplings from patients with meningeal metastases are awaited, the existence of alternative or resistance mechanisms that may be activated in response to the inhibition of the BRAF-driven pathway represents a clear indication that a combination of targeted compounds should be planned for the treatment of melanoma beyond disease progression.

## **Consent**

Written informed consent was obtained from the patient’s husband for publication of this case report and any accompanying images. A copy of the written consent is available for review by the Editor-in-Chief of this journal.

## **Competing interests**

The authors declare that they have no competing interests, with the exception of PAA, who stood on the Advisory Board of Bristol Myers Squibb, Merck Sharp & Dohme, Roche-Genentech, GSK and Amgen and received honoraria from Bristol Myers Squibb, Merck Sharp & Dohme, Roche-Genentech; and SS, who received honoraria from Bristol Myers Squibb.

## **Authors’ contributions**

ES, EDM, PA, VM and PAA performed all the clinical analyses. FF, SS and GP performed cytological and histopathological classifications. FS and SL performed the radiological and positron emission tomography scan evaluation. ES, EDM and GP helped to draft the manuscript. ES and NM participated in the design of the study. PAA conceived of the study and drafted the manuscript. All authors read and approved the final manuscript.
